# Improving the Robustness of Entangled States by Basis Transformation

**DOI:** 10.3390/e21010059

**Published:** 2019-01-13

**Authors:** Xin-Wen Wang, Shi-Qing Tang, Yan Liu, Ji-Bing Yuan

**Affiliations:** 1 College of Physics and Electronic Engineering, Hengyang Normal University, Hengyang 421002, China; 2 Hunan Provincial Key Laboratory of Intelligent Information Processing and Application, Hengyang Normal University, Hengyang 421002, China; 3 Key Laboratory of Low-Dimensional Quantum Structures and Quantum Control of Ministry of Education, Hunan Normal University, Changsha 410081, China

**Keywords:** entanglement robustness, cat-like states, local Pauli noises, robustness-enhancement method, basis transformation

## Abstract

In the practical application of quantum entanglement, entangled particles usually need to be distributed to many distant parties or stored in different quantum memories. In these processes, entangled particles unavoidably interact with their surrounding environments, respectively. We here systematically investigate the entanglement-decay laws of cat-like states under independent Pauli noises with unbalanced probability distribution of three kinds of errors. We show that the robustness of cat-like entangled states is not only related to the overall noise strength and error distribution parameters, but also to the basis of qubits. Moreover, we find that whether a multi-qubit state is more robust in the computational basis or transversal basis depends on the initial entanglement and number of qubits of the state as well as the overall noise strength and error distribution parameters of the environment. However, which qubit basis is conductive to enhancing the robustness of two-qubit states is only dependent on the error distribution parameters. These results imply that one could improve the intrinsic robustness of entangled states by simply transforming the qubit basis at the right moment. This robustness-improving method does not introduce extra particles and works in a deterministic manner.

## 1. Introduction

Quantum entanglement, a typical non-classical correlation between quantum systems, is at the center of quantum information science [1,2,3,4]. Most quantum communication, computation, and metrology protocols are based on quantum entanglement [5,6,7]. However, entanglement is very fragile due to the unavoidable interactions between the entangled systems and their surrounding environments [8]. Local system–environment interaction usually happens in the scenarios related to quantum communication and distributed quantum computation where entangled particles are far apart [9,10]. Entanglement decay, resulting from environment-induced decoherence, will negatively affect the quality of related quantum information processing tasks [10,11,12]. The problem on how to harness entanglement of quantum systems against the detrimental effects of the environment is of utmost importance within the vast domain of studies of quantum entanglement, since it is directly connected to the applications of quantum entanglement [13,14]. A variety of strategies have been proposed for protecting quantum entanglement [15,16,17,18,19,20,21,22,23,24,25,26,27,28,29,30,31,32,33].

Entanglement distillation is a prevalent way to improve the entanglement of distant particles in mixed states [33,34,35,36]. Except for some special scenarios [37,38,39], the initial fidelity (relative to maximally entangled states) or entanglement degree of the source states (input states) in an entanglement distillation protocol must be larger than a threshold [33,34,35,36]. Generally, the higher degree of entanglement the input states have, the higher degree of entanglement the output states have, or the higher the distillation efficiency is, provided that the initial entanglement of the source states meets the threshold requirement. Quantum filtration methods could be used to probabilistically increase the fidelity of source states with particular structures such that it meets the threshold required for an entanglement distillation protocol [40,41]. However, quantum filtering operations may decrease the final distillation efficiency in the case where the entanglement degree of the source states exceeds the required threshold [42]. Enhancing the intrinsic robustness of entangled states is thus of importance [43,44,45,46,47].

Pauli noise is a kind of typical noise for qubits [8,9]. The action of a general Pauli noise on any state ρ of a qubit can be formulated as

(1) $$\begin{array}{c} E\left(\rho \right)=\left(1-\frac{p}{2}\right)\rho +\frac{p}{2}\left(a_{x}\sigma_{x}\rho \sigma_{x}+a_{y}\sigma_{y}\rho \sigma_{y}+a_{z}\sigma_{z}\rho \sigma_{z}\right), \end{array}$$

where $\sigma_{x}$, $\sigma_{y}$, and $\sigma_{z}$ are the conventional three Pauli matrices in the computational basis {|0〉,|1〉}. Parameters $0⩽a_{x},a_{y},a_{z}⩽1$ satisfy the normalization condition $a_{x}+a_{y}+a_{z}=1$. Probability 0⩽p⩽1 measures the noise strength and gives also a convenient parametrization of time: p=0 refers to the initial time t=0 and p=1 refers to the asymptotic t→∞ limit. Note that the 1/2 factors in Equation (1) are such that an exact fully dephasing channel appears at p=1 and $a_{z}=1$. The particular examples of single-qubit Pauli noises are the depolarizing, phase-flip, bit-flip, and bit-phase-flip channels, which correspond to $a_{x}=a_{y}=a_{z}=1/3$, $a_{z}=1$ ($a_{x}=a_{y}=0$), $a_{x}=1$ ($a_{y}=a_{z}=0$), and $a_{y}=1$ ($a_{x}=a_{z}=0$), respectively. A depolarizing channel describes that the qubit is subjected to bit-flip ($\sigma_{x}$), phase-flip ($\sigma_{z}$), and bit-phase-flip ($\sigma_{y}$) errors with the same probability. In the other three noise channels, only one type of error happens. An entangled state usually displays different robustness in different noise channels [48,49,50,51]. The robustness of different entangled states, even with the same initial entanglement, may be different in the same noise channel [44,47,52].

In many cases, the above-mentioned three types of errors may happen with different probabilities [12,47]. Two examples are listed bellow. Consider that several physical qubits are used to encode one logical qubit. Errors acting on the individual qubits lead to the populations outside the logical subspace. However, active quantum error correction allows one to correct certain errors, while other errors may lead to an error at the logical level. It has been shown that, for a repetition code capable of correcting bit-flip errors, depolarizing noise at the physical level leads to effective Pauli noise with a preferred direction at the logical level [53]. Generally, depolarizing noise acting on physical qubits encoding a logical qubit could be modeled as effective Pauli noise, with unbalanced probability distribution for the three errors, acting on the logical qubit [53]. Another example is provided by thermal baths with infinite temperature, where the decoherence of a qubit can be described by a Pauli map with $a_{x}=a_{y}\neq a_{z}$ [12,44].

In this paper, we investigate the disentanglement features and robustness of *n*-qubit (n⩾2) cat-like entangled states under the local (independent) Pauli channels where the probabilities of three kinds of errors occurring are not the same. We mainly discuss three issues. One is how the error distribution parameters ($a_{x},a_{y},a_{z}$) affect the residual entanglement. Another one is whether the initial entanglement degree of an entangled state has influence on its decay law. The third one is how the qubit basis impacts the entanglement-robustness of cat-like states. What we are most interested in is under which qubit basis (computational or transversal basis) an entangled state is more robust in a noisy environment. We shall show that the answer depends not only on the noise parameters, but also on the entanglement degree of the original entangled state and the number of involved qubits. More interestingly, two-qubit entangled states may exhibit the opposite phenomenon to multi-qubit entangled states.

## 2. Robustness of Cat-Like States and Its Enhancement Method

When *n* Pauli noises independently act on *n* qubits of any state ϱ, the composite *n*-qubit map M is given by the single-qubit map composition

(2) $$M\left(\varrho \right)=E_{1}⊗E_{2}⊗\cdots E_{k}⊗\cdots E_{n}\left(\varrho \right),$$

where $E_{k}$, with 1⩽k⩽n, corresponds to map (1) acting on the *k*th qubit. We shall discuss the entanglement-robustness of different *n*-qubit cat-like states under the map M. The entanglement between any two parts of a decohered state, e.g., one qubit versus the rest, will be measured by the negativity [54,55]. Following Ref. [55], the negativity can be defined as

(3) $$N\left(\varrho \right)=-2\lambda_{\min},$$

where $\lambda_{\min}$ is the sum of all minus eigenvalues of the partial transpose of the state ϱ. Note that, when the two parts are separable, one should let $\lambda_{\min}\equiv 0$. It will be shown that the robustness of an entangled state is dependent on the form of qubit basis, the number of qubits *n*, the noise strength *p*, and the error distribution parameters ($a_{x},a_{y},a_{z}$). Moreover, two-qubit entangled states express different decoherence behaviours from multi-qubit entangled states.

### 2.1. Robustness of Cat-Like States in the Computational Basis

For the initial *n*-qubit (n⩾2) cat-like states

(4) $$\begin{array}{c} |\Phi_{n}^{+}{\rangle =\alpha |0\rangle }^{⊗n}+\beta {|1\rangle }^{⊗n} \end{array}$$

with $\alpha^{2}+\beta^{2}=1$ (for simplicity, α and β are assumed to be real), the amount of entanglement between any two partitions is

(5) $$N_{i}=2\left|\alpha \beta \right|.$$

When $\left|\alpha \right|=\left|\beta \right|=1/\sqrt{2}$, $N_{i}=1$ and the *n*-qubit states in Equation (4) are maximally entangled states. If |α|≠|β|, $N_{i}<1$ and these states are partially entangled pure states. The decohered states $M(|\Phi_{n}^{+}\rangle )$ are X states [46,56]. For any bipartition of an *n*-qubit X matrix, its partial-transpose is still an X matrix with the same dimension. Calculation of the eigenvalues of a $2^{n}\times 2^{n}$-dimensional X matrix is essentially equivalent to diagonalizing $2^{n-1}$ matrices of dimension 2×2. With these features, one can calculate, in a straightforward way, the negativity N of any bipartition “one qubit versus the rest” of $M(|\Phi_{n}^{+}\rangle )$.

For the phase-flip channel $M^{PF}$, i.e., $a_{z}=1$, we have

(6) $$N_{\left(a_{z}=1\right)}={\left(1-p\right)}^{n}N_{i}.$$

Obviously, $N_{\left(a_{z}=1\right)}$ decays exponentially with *n*, as ${\left(1-p\right)}^{n}$. In fact, all the entanglement in the state $|\Phi_{n}^{+}\rangle$ under $M^{PF}$ decays (at slowest) exponentially with *n*. This implies that the state $|\Phi_{n}^{+}\rangle$ is very fragile to the phase-flip noise.

As to a non-pure phase-damping channel, i.e., $a_{z}<1$, the negativity of any bipartition “one qubit versus the rest” of $M(|\Phi_{n}^{+}\rangle )$ can also be analytically calculated, which is given by (Appendix A)

(7) $$\begin{array}{ccc} N & = & \sum_{u=0}^{\left\lfloor \frac{n-1}{2}\right\rfloor }\left(\begin{array}{c} n-1\\ u \end{array}\right)\left[\max\left(0,G_{u}-F_{u+1}\right)+\max\left(0,H_{u}-F_{u}\right)\right], \end{array}$$

where $\left\lfloor \frac{n-1}{2}\right\rfloor =\left(n-1\right)/2$, for *n* being odd, or $\lfloor \frac{n-1}{2}\rfloor =n/2-1$, for *n* being even, and

(8) $$\;\begin{array}{cc} F_{u} & =A^{n-u}B^{u}+A^{u}B^{n-u}, \end{array}$$

(9) $$\begin{array}{cc} G_{u} & ={\left[{\left(A^{n-u-1}B^{u+1}-A^{u+1}B^{n-u-1}\right)}^{2}\left(1-N_{i}^{2}\right)+{\left(C^{n-u}D^{u}+C^{u}D^{n-u}\right)}^{2}N_{i}^{2}\right]}^{1/2}, \end{array}$$

(10) $$\begin{array}{cc} H_{u} & ={\left[{\left(A^{n-u}B^{u}-A^{u}B^{n-u}\right)}^{2}\left(1-N_{i}^{2}\right)+{\left(C^{n-u-1}D^{u+1}+C^{u+1}D^{n-u-1}\right)}^{2}N_{i}^{2}\right]}^{1/2}, \end{array}$$

(11) $$\;\begin{array}{cc} A & =1-\frac{p}{2}+\frac{p}{2}a_{z}, \end{array}$$

(12) $$\;\begin{array}{cc} B & =\frac{p}{2}a_{x}+\frac{p}{2}a_{y}, \end{array}$$

(13) $$\;\begin{array}{cc} C & =1-\frac{p}{2}-\frac{p}{2}a_{z}, \end{array}$$

(14) $$\;\begin{array}{cc} D & =\frac{p}{2}a_{x}-\frac{p}{2}a_{y}. \end{array}$$

Obviously, the negativity of the decohered state is dependent on both channel parameters ($a_{x},a_{y},a_{z},p$) and the amount of entanglement of the initial state ($N_{i}$). In the following, we shall show some interesting results.

**Figure 1:**
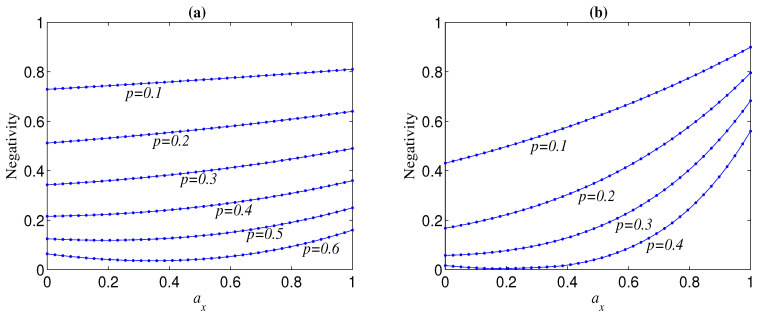
Negativity of decohered state $M(|\Phi_{n}^{+}\rangle )$, as a function of $a_{x}$, for different noise strength *p*, where $a_{y}=0$ and $N_{i}=1$. (**a**) n=3; (**b**) n=8.

We first discuss the case $N_{i}=1$, i.e., the original state in Equation (4) is a standard Greenberger–Horne–Zeilinger (GHZ) state (n⩾3) or Bell state (n=2). When the initial state is a standard GHZ, the variation tendency of negativity N of the decohered state with error distribution parameters ($a_{x},a_{y},a_{z}$) is related to the overall noise strength *p*. For a given $a_{y}$, the increase (decrease) in $a_{x}$ will always lead to increase (decrease) in the negativity N when *p* is small; however, N may first slowly decrease and then increase (even rapidly) with the increase of $a_{x}$ when *p* exceeds a threshold (see, e.g., Figure 1). It can be seen from Figure 1 that the threshold of *p* is usually related to *n*, and the larger *n*, the smaller threshold, and that, for a given *n*, the larger *p* is, the larger $a_{x}$ the knee point of N happens at. Moreover, the larger *n* is, N is more dependent on $a_{x}$, i.e., it is more sensitive to the change of $a_{x}$. Overall, for given *p* and $a_{y}$, the residual entanglement of the decohered GHZ state tends to a maximum when $a_{x}\to 1-a_{y}$. These phenomena imply that the standard GHZ states under the computational basis are the most robust against the noise of $\sigma_{x}$ direction (which is vertical to the qubit-basis direction in the Bloch sphere), and that the variation tendency of the negativity of a decohered state with the weight of the $\sigma_{x}$-directional noise is slightly modulated by the overall noise strength.

**Figure 2:**
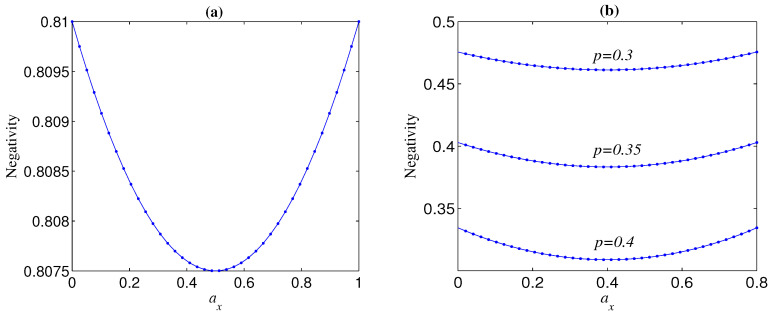
Negativity of decohered state $M(|\Phi_{2}^{+}\rangle )$, as a function of $a_{x}$, for different noise strength *p*, where $N_{i}=1$. (**a**) $a_{y}=0,\;p=0.1$; (**b**) $a_{y}=0.2$.

The Bell state displays different decoherence behaviour from GHZ states. For any given *p* and $a_{y}$, $N_{\left(n=2\right)}$ is not monotonous with $a_{x}$ and the knee point always happens at $a_{x}=\left(1-a_{y}\right)/2$, though $a_{x}$ has a very slight influence on it (see, e.g., Figure 2). Furthermore, $N_{\left(n=2\right)}$ takes a maximum when $a_{x}$ equals to zero or $1-a_{y}$. In other words, when $a_{x}<\left(1-a_{y}\right)/2$ ($a_{x}>\left(1-a_{y}\right)/2$), the smaller (larger) $a_{x}$, the larger $N_{\left(n=2\right)}$. These results indicate that when the weights of the phase-flip error and bit-flip error are more different, the Bell state is more robust.

**Figure 3:**
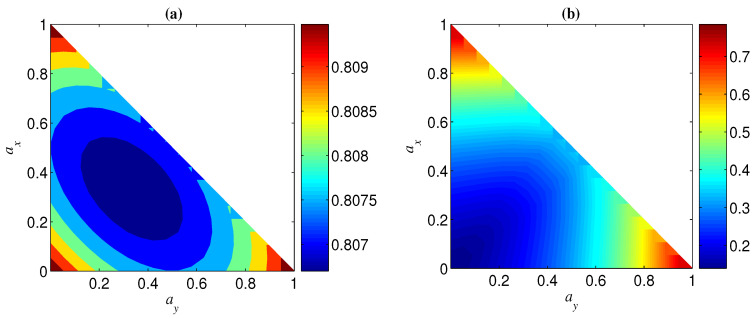
Negativity as a function of $a_{x}$ and $a_{y}$ for initial maximally entangled pure state $|\Phi_{n}^{+}\rangle$ under local decoherence. (**a**) n=2,p=0.1; (**b**) n=9,p=0.2.

**Figure 4:**
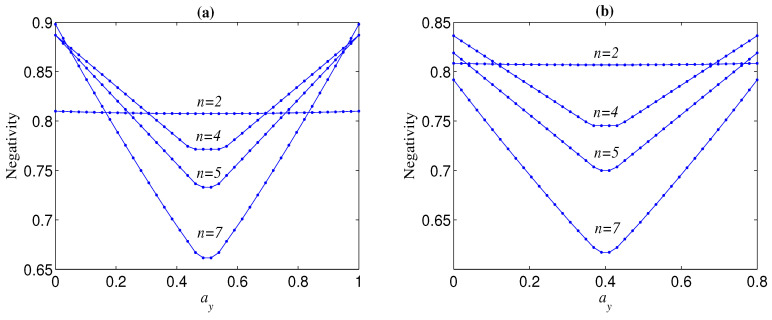
Negativities as functions of $a_{y}$ for different initial pure states $|\Phi_{n}^{+}\rangle$ with $N_{i}=1$ under local decoherence, where p=0.1. (**a**) $a_{z}=0$; (**b**) $a_{z}=0.2$.

One can check another interesting phenomenon that $a_{y}$ has a similar influence with $a_{x}$ on N (see, e.g., Figure 3). This phenomenon could be partly understood from the fact that both eigenvectors of noisy operators $\sigma_{y}$ and $\sigma_{x}$ are vertical to basis vectors |0〉 and |1〉 in the Bloch sphere. Moreover, for any given $a_{z}$, N always takes a minimum when $a_{y}=\left(1-a_{z}\right)/2$, i.e., $a_{y}=a_{x}$ (see Figure 3). This conclusion can be directly obtained from Equation (7) when n=2. As to n>2, it could be explained as follows. The standard GHZ states under the computational basis are the most fragile to the phase-flip error and the most robust against the bit-flip error (will be shown later). On the other hand, the noisy operator $\sigma_{y}$ has both phase-flip and bit-flip actions. Thus, there is a trade-off between the negative effect and positive effect of the weight of the phase-bit-flip error. For a given $a_{z}$, the relation between N and $a_{y}$ is clearly shown in Figure 4. It can be seen from Figure 4 that the influence degree of $a_{y}$ on N is symmetric about $a_{y}=\left(1-a_{z}\right)/2$ for both Bell and GHZ states, and N takes a maximum when $a_{y}$ equals to zero or $1-a_{z}$.

**Figure 5:**
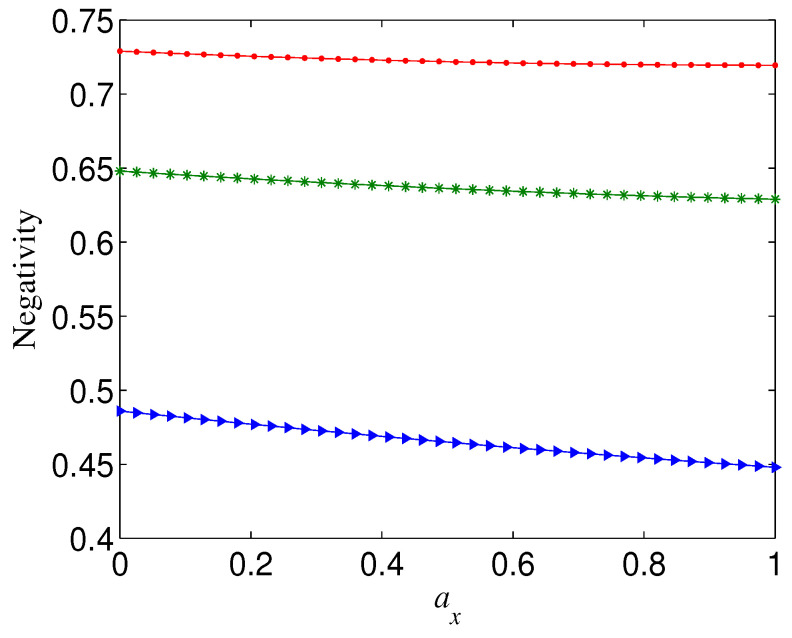
Negativity of decohered state $M(|\Phi_{2}^{+}\rangle )$, as a function of $a_{x}$, for $N_{i}=0.9$ (red dots), 0.8 (green stars), or 0.6 (blue triangles), where $a_{y}=0,\;p=0.1$.

**Figure 6:**
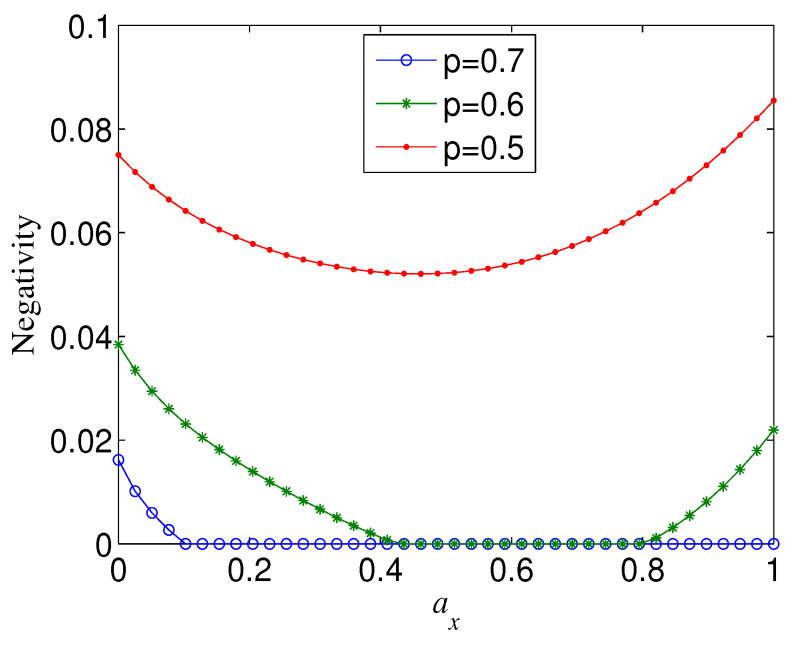
Negativity of decohered state $M(|\Phi_{3}^{+}\rangle )$ versus noisy parameter $a_{x}$, where $a_{y}=0,\;N_{i}=0.6$.

The influence of the channel parameters on the negativity N of the decohered state is also relative to the amount of entanglement $N_{i}$ of the initial state (4). As examples, Figure 5 and Figure 6 show that, when $N_{i}<1$ (i.e., |α|≠|β|), N (for a given $a_{y}$) exhibits different dynamic behaviour with the variation of $a_{x}$ from the case $N_{i}=1$ (i.e., |α|=|β|) for n=2,3, respectively. Specifically, if $N_{i}<1$, $N_{\left(n=2\right)}$ tends to a minimum when $a_{x}\to 1-a_{y}$ (e.g., $a_{y}=0$), in contrast to the case $N_{i}=1$ where $N_{\left(n=2\right)}$ tends to a maximum when $a_{x}\to 1-a_{y}$. In the case $N_{i}<1$ (e.g., $N_{i}=0.6$), $N_{\left(n=3\right)}$ takes a maximum at $a_{x}=0$ when *p* exceeds a threshold. One can check that, for $N_{i}<1$, $N_{\left(n⩾4\right)}$ could also take a maximum at $a_{x}=0$ when *p* is very large. Note that, if $N_{i}=1$, N always reaches a maximum when $a_{x}$ increases to $1-a_{y}$ for any given *p* and $a_{y}$, as mentioned above. The difference of the decay behaviours of partially entangled states and maximally ones may be partly understood from the fact that the two distinguishable product states (${|0\rangle }^{⊗n}$ and ${|1\rangle }^{⊗n}$) are superposed with unequal weights in the former and equal weight in the latter.

**Figure 7:**
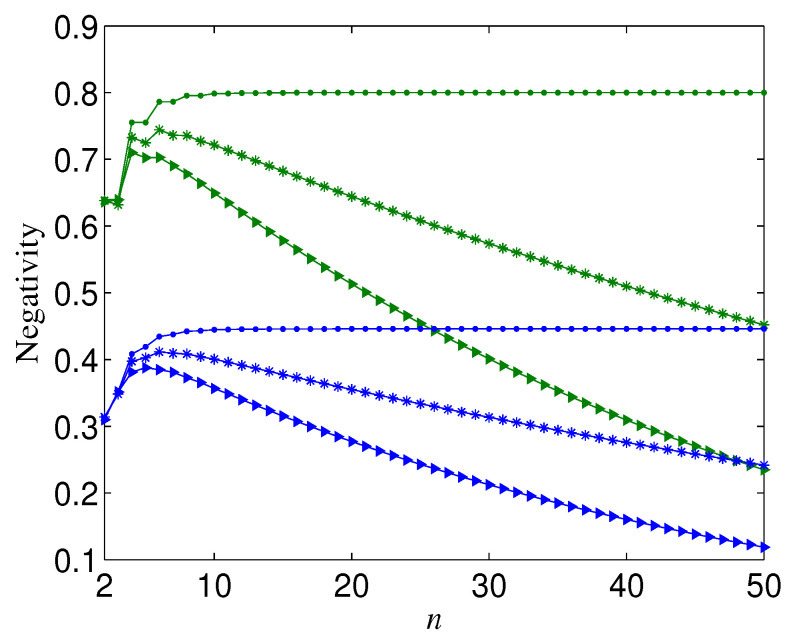
Negativity (N) versus *n*, where p=0.2. Green lines correspond to $N_{i}=1$; blue lines correspond to $N_{i}=0.6$. Dots correspond to $a_{x}=1,\;a_{y}=0$; stars correspond to $a_{x}=0.95,\;a_{y}=0$; triangles correspond to $a_{x}=0.9,\;a_{y}=0.1$.

**Figure 8:**
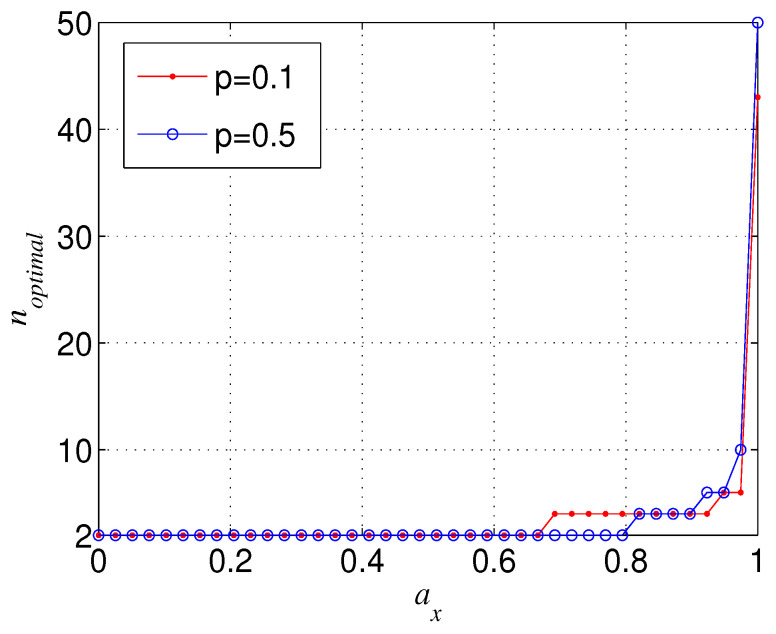
The optimal *n* ($n_{optimal}$) that maximizes N for different $a_{x}$, where $N_{i}=1$ and $a_{y}=0$.

From Figure 4a, we can also observe that the more larger *n*, N is more sensitive to the change of $a_{y}$ (or $a_{x}$), which leads to the fact that when $a_{y}$ tends to zero ($a_{y}$ tends to one) and $a_{x}$ tends to one ($a_{x}$ tends to zero), $N_{\left(n=M\right)}$ could be larger than $N_{\left(n=L\right)}$ with M>L⩾2. Figure 7 shows the trend of the negativity N with the variation of number of qubits *n* when $a_{y}$ is near to zero ($a_{y}$ is near to one) and $a_{x}$ is near to one ($a_{x}$ is near to zero). It can be seen from Figure 7 that the total variation tendency of N with *n* is independent from the initial entanglement $N_{i}$. Figure 7 also implies that the optimal *n* that maximizes the bipartite entanglement of the decohered *n*-qubit state is dependent on $a_{x}$ ($a_{y}$) when $a_{y}\to 0$ ($a_{x}\to 0$). In Figure 8, we give the aforementioned optimal *n* for different $a_{x}$ with $a_{y}=0$. From Figure 8, we can deduce that, when $a_{x}$ is infinitely close to one, the optimal *n* always tends to infinity.

In fact, when $a_{x}=1$, the channel map M reduces to the bit-flip channel $M^{\mathrm{BF}}$, and we can analytically obtain

(15) $$\begin{array}{c} E\left(M^{\mathrm{BF}}(|\Phi_{n}^{+}\rangle )\right)⩾E\left(M^{\mathrm{BF}}(|\Phi_{n-1}^{+}\rangle )\right)⩾\cdots ⩾E\left(M^{\mathrm{BF}}(|\Phi_{2}^{+}\rangle )\right), \end{array}$$

where E(·) is an arbitrary entanglement measure. Note that this result is independent from the noise strength *p*. It can be understood as follows. A single-qubit $\sigma_{x}$ measurement on $|\Phi_{n}^{+}\rangle$ leaves the system in state $|\Phi_{n-1}^{+}\rangle ⊗|+\rangle$ or $|\Phi_{n-1}^{-}\rangle ⊗|-\rangle$, with $|\pm \rangle =(|0\rangle +|1\rangle )/\sqrt{2}$ being the eigenstates of $\sigma_{x}$ and $|\Phi_{n-1}^{-}{\rangle =\alpha |0\rangle }^{⊗n-1}-\beta {|1\rangle }^{⊗n-1}$. Similarly, since it commutes with $M^{\mathrm{BF}}$, a $\sigma_{x}$ measurement on $\rho_{n}^{+}=M^{\mathrm{BF}}(|\Phi_{n}^{+}\rangle )$ leaves the system in $\rho_{n-1}^{+}⊗|+\rangle \langle +|$ or $\rho_{n-1}^{-}⊗|-\rangle \langle -|$, with $\rho_{n-1}^{-}=M^{\mathrm{BF}}(|\Phi_{n-1}^{-}\rangle )$. Furthermore, it is immediate to see that $\rho_{n-1}^{+}⊗|+\rangle \langle +|$ and $\rho_{n-1}^{-}⊗|-\rangle \langle -|$ are local-unitarily equivalent. Thus, we can say $E\left(\rho_{n}^{+}\right)⩾E\left(\rho_{n-1}^{+}\right)$. Iterating this reasoning n−2 times and, for ease of notation, omitting the tensor-product factors, one can obtain Equation (15). For specific calculation of the negativity given in Equation (7), one can omit the second term in each square bracket because $H_{u}$ is impossible to be larger than $F_{u}$ when $a_{x}=1$.

### 2.2. Robustness of Cat-Like States in the Transversal Basis

By local Hadamard-gate rotation H=|+〉〈0|+|−〉〈1|, the cat-like state $|\Phi_{n}^{+}\rangle$ can be transformed into the transversal form, i.e.,

(16) $$\begin{array}{c} |\Phi_{n}^{+T}\rangle =H^{⊗n}|\Phi_{n}^{+}{\rangle =\alpha |+\rangle }^{⊗n}+\beta {|-\rangle }^{⊗n}. \end{array}$$

The transversal state $|\Phi_{n}^{+T}\rangle$ is local-unitarily equivalent to $|\Phi_{n}^{+}\rangle$, and thus possesses the same amount and type of entanglement as $|\Phi_{n}\rangle$. However, it will be shown that $|\Phi_{n}^{+T}\rangle$ may display a very different decoherence behaviour from $|\Phi_{n}^{+}\rangle$ in the same noisy environment.

The decohered state $M(|\Phi_{n}^{+T}\rangle )$ is also an X state under the transversal qubit-basis {|+〉,|−〉}. In the local bit-flip channels, i.e., $a_{x}=1$, the negativity $N^{T}$ of any bipartition “one qubit versus the rest” of $M(|\Phi_{n}^{+T}\rangle )$ is the same as $N_{\left(a_{z}=1\right)}$ in Equation (6). For $a_{x}<1$, $N^{T}$ has the same form as Equation (7), but with A,B,C,D being replaced, respectively, by $A^{\prime },B^{\prime },C^{\prime },D^{\prime }$, which are given by

(17) $$\begin{array}{cc} A^{\prime } & =1-\frac{p}{2}+\frac{p}{2}a_{x}, \end{array}$$

(18) $$\begin{array}{cc} B^{\prime } & =\frac{p}{2}a_{z}+\frac{p}{2}a_{y}, \end{array}$$

(19) $$\begin{array}{cc} C^{\prime } & =1-\frac{p}{2}-\frac{p}{2}a_{x}, \end{array}$$

(20) $$\begin{array}{cc} D^{\prime } & =\frac{p}{2}a_{z}-\frac{p}{2}a_{y}. \end{array}$$

The dependency relationship of $N^{T}$ on $a_{x}$ ($a_{z}$) is different from that of N on $a_{x}$ ($a_{z}$). If $a_{z}=1$, the Pauli channel M reduces to the phase-flip channel $M^{\mathrm{PF}}$, the inequalities in Equation (15) also hold for states $M^{PF}(|\Phi_{n}^{+T}\rangle )$. As a matter of fact, the role of $a_{z}$ in $N^{T}$ is the same as that of $a_{x}$ in N. The essential cause is that the action of operator $\sigma_{z}$ on the basis states {|+〉,|−〉} is the same as $\sigma_{x}$ on {|0〉,|1〉}, and vice versa. Thus, the result about the influence of $a_{x}$ on the residual entanglement of $|\Phi_{n}^{+}\rangle$ in the noisy environment described by M shown in the former subsection is also applicable for the influence of $a_{z}$ on the residual entanglement of $|\Phi_{n}^{+T}\rangle$.

The impact of $a_{y}$ on both $M(|\Phi_{n}^{+T}\rangle )$ and $M(|\Phi_{n}^{+}\rangle )$ is the same. This is due to the fact that $\sigma_{y}\left|0\rangle \langle 0\right|\sigma_{y}=\sigma_{y}\left|+\rangle \langle +\right|\sigma_{y}$, $\sigma_{y}\left|1\rangle \langle 1\right|\sigma_{y}=\sigma_{y}\left|-\rangle \langle -\right|\sigma_{y}$, and $\sigma_{y}\left|0\rangle \langle 1\right|\sigma_{y}=\sigma_{y}\left|+\rangle \langle -\right|\sigma_{y}$, that is, the operator $\sigma_{y}$ has the same effect on a qubit under both bases. If $a_{y}=1$, i.e., the channel M reduces to the bit-phase-flip channel $M^{\mathrm{BPF}}$, the inequalities in Equation (15) hold for both states $M^{BPF}(|\Phi_{n}^{+}\rangle )$ and $M^{BPF}(|\Phi_{n}^{+T}\rangle )$. It can be understood as follows. A single-qubit $\sigma_{y}$ measurement on $|\Phi_{n}^{+}\rangle$ leaves the system in state $|\Phi_{n-1}^{\prime -}{\rangle ⊗|+\rangle }_{y}$ or $|\Phi_{n-1}^{\prime +}{\rangle ⊗|-\rangle }_{y}$, with ${|\pm \rangle }_{y}=(|0\rangle \pm i|1\rangle )/\sqrt{2}$ being the eigenstates of $\sigma_{y}$ and $|\Phi_{n-1}^{\prime \pm }{\rangle =\alpha |0\rangle }^{⊗n-1}\pm i\beta {|1\rangle }^{⊗n-1}$. Note that $|\Phi_{n-1}^{\prime \pm }\rangle$ are local-unitarily equivalent to $|\Phi_{n-1}^{+}\rangle$. Similarly, since it commutes with $M^{\mathrm{BPF}}$, a $\sigma_{y}$ measurement on $\rho_{n}^{+}=M^{\mathrm{BPF}}(|\Phi_{n}^{+}\rangle )$ leaves the system in $\rho_{n-1}^{\prime -}{⊗|+\rangle }_{y}\langle +|$ or $\rho_{n-1}^{\prime +}{⊗|-\rangle }_{y}\langle -|$, with $\rho_{n-1}^{\prime \pm }=M^{\mathrm{BPF}}(|\Phi_{n-1}^{\prime \pm }\rangle )$. Furthermore, it is immediate to see that $\rho_{n-1}^{\prime \pm }$ are local-unitarily equivalent to $\rho_{n-1}^{+}$. Thus, we can say $E\left(\rho_{n}^{+}\right)⩾E\left(\rho_{n-1}^{+}\right)$. Iterating this reasoning n−2 times and, for ease of notation, omitting the tensor-product factors, one can obtain Equation (15) for states $M^{BPF}(|\Phi_{n}^{+}\rangle )$. In the same vein, we can also prove the inequalities in (15) for states $M^{BPF}(|\Phi_{n}^{+T}\rangle )$. Specifically, a single-qubit $\sigma_{y}$ measurement on $|\Phi_{n}^{+T}\rangle$ will leave the system in state $|\Phi_{n-1}^{\prime +T}{\rangle ⊗|+\rangle }_{y}$ or $|\Phi_{n-1}^{\prime -T}{\rangle ⊗|-\rangle }_{y}$, with $|\Phi_{n-1}^{\prime \pm T}\rangle =e^{\mp \pi i/4}{\alpha |+\rangle }^{⊗n-1}+e^{\pm \pi i/4}\beta {|-\rangle }^{⊗n-1}$ being local-unitarily equivalent to $|\Phi_{n-1}^{+T}\rangle$. Then, a $\sigma_{y}$ measurement on $\rho_{n}^{+T}=M^{\mathrm{BPF}}(|\Phi_{n}^{+T}\rangle )$ will leave the system in $\rho_{n-1}^{\prime +T}{⊗|+\rangle }_{y}\langle +|$ or $\rho_{n-1}^{\prime -T}{⊗|-\rangle }_{y}\langle -|$, with $\rho_{n-1}^{\prime \pm T}=M^{\mathrm{BPF}}(|\Phi_{n-1}^{\prime \pm T}\rangle )$ being local-unitarily equivalent to $\rho_{n-1}^{+T}$. Thus, we can conclude $E\left(\rho_{n}^{+T}\right)⩾E\left(\rho_{n-1}^{+T}\right)$. Repeating the process above n−2 times, one can verify Equation (15) for states $M^{BPF}(|\Phi_{n}^{+T}\rangle )$.

It should be pointed out again that $\sigma_{y}$ plays the same role as $\sigma_{x}$ in states $M(|\Phi_{n}^{+}\rangle )$ (see Figure 3 and Figure 4) and as $\sigma_{z}$ in states $M(|\Phi_{n}^{+T}\rangle )$. This may be partly understood from the fact that the eigenvectors of both noisy operators $\sigma_{y}$ and $\sigma_{x}$ are transversal to the qubit basis {|0〉,|1〉}, and the eigenvectors of both $\sigma_{y}$ and $\sigma_{z}$ are transversal to the qubit basis {|+〉,|−〉}.

### 2.3. Enhancing the Robustness of Cat-Like States by Basis Transformation

According to the discussion above, we obtain that, under the local Pauli channels with unbalanced probability distribution for three kinds of errors, the entanglement-robustness of cat-like states are generally related to the qubit basis. This implies that one can enhance the robustness of entangled states by transforming the qubit basis according to preestimated channel features. In this subsection, we investigate in what conditions the basis {|0〉,|1〉} is better than {|+〉,|−〉}, or otherwise. This can be achieved by comparing the negativity $N^{T}$ of $M(|\Phi_{n}^{+T}\rangle )$ and the negativity N of $M(|\Phi_{n}^{+}\rangle )$. If the difference

(21) $$\Delta N=N^{T}-N$$

is larger than zero, it indicates that $|\Phi_{n}^{+T}\rangle$ is more robust than $|\Phi_{n}^{+}\rangle$. If ΔN<0, it means $|\Phi_{n}^{+}\rangle$ is more robust than $|\Phi_{n}^{+T}\rangle$. As mentioned above, the noisy operator $\sigma_{y}$ has the same effect on a qubit under both bases {|+〉,|−〉} and {|0〉,|1〉}; thus, ΔN≡0 when $a_{y}=1$. Then, we only need to discuss the case $a_{y}<1$.

**Figure 9:**
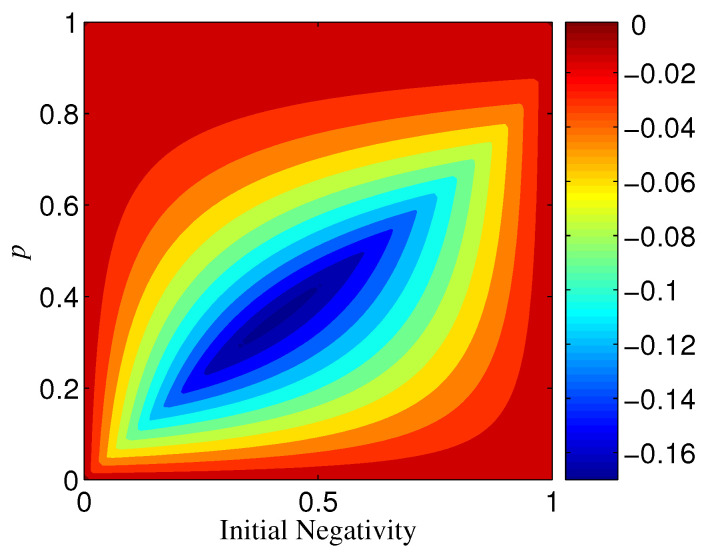
Variation of ΔN with different *p* and $N_{i}$ when $a_{z}=1$ and n=2.

Let us begin by the phase-flip channel $a_{z}=1$. For n=2, it can be directly calculated that ΔN=0 when $N_{i}=1$; however, ΔN⩽0 when $N_{i}<1$ (see Figure 9). Thus, we can conclude that the two-qubit maximally entangled state (|α|=|β|) has the same robustness under both qubit bases {|+〉,|−〉} and {|0〉,|1〉}, but two-qubit partially entangled states (|α|≠|β|) are more robust in the basis {|0〉,|1〉} than in the basis {|+〉,|−〉}. If consider the possible fact that it is very difficult to make |α| be strictly equal to |β| in experiments, we could say that the basis {|0〉,|1〉} is superior to the basis {|+〉,|−〉} for two-qubit state distribution or storage.

**Figure 10:**
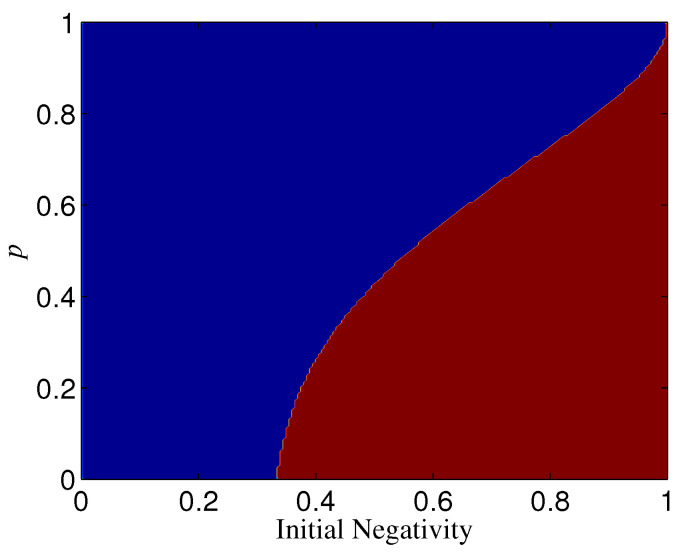
The sign (positive or minus) of $\Delta N_{\left(n=3\right)}$ for different *p* and $N_{i}$, where $a_{z}=1$. The blue region denotes ΔN<0 and the red region denotes ΔN>0.

**Figure 11:**
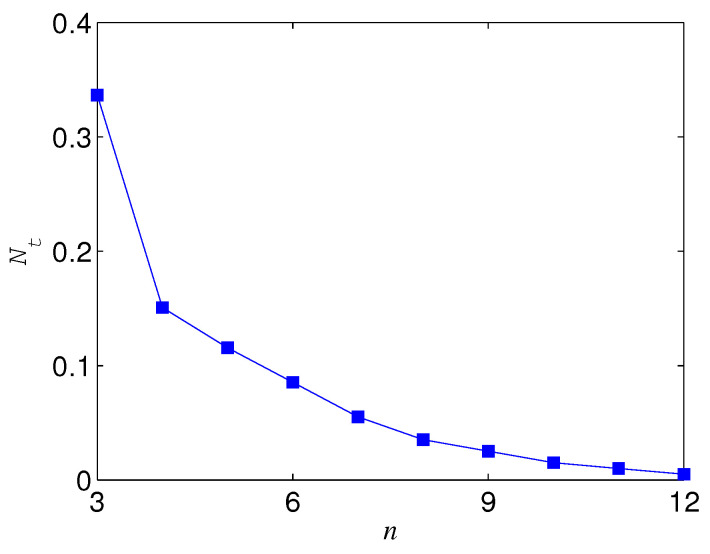
$N_{t}$ versus *n*, where $a_{z}=1$.

**Figure 12:**
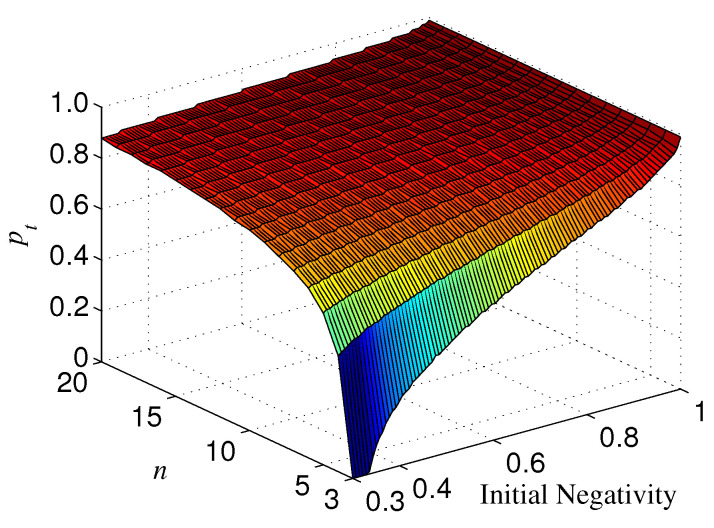
$p_{t}$ for different $N_{i}$ and *n*, where $a_{z}=1$.

The results for n⩾3 are not the same as that for n=2. If $N_{i}=1$, ΔN is always positive for n⩾3, which indicates that the basis {|+〉,|−〉} overmatches {|0〉,|1〉} in keeping the entanglement of standard GHZ states. If $N_{i}<1$, however, the sign (positive or minus) of ΔN is dependent on $N_{i}$, *n*, and *p*. When $N_{i}$ is less than a threshold $N_{t}$, ΔN<0, which means that $|\Phi_{n}^{+}\rangle$ is more robust than $|\Phi_{n}^{+T}\rangle$ (see, e.g., Figure 10). When $N_{t}<N_{i}<1$, ΔN could also be minus as long as *p* is larger than a threshold $p_{t}$ depending on $N_{i}$ (see Figure 10). Generally, for a given *n*, the larger $N_{i}$, the larger $p_{t}$. In addition, the larger *n*, the less $N_{t}$; when *n* is very large, $N_{t}\to 0$. $N_{t}$ with different *n* is given in Figure 11. We plot $p_{t}$ as a function of $N_{i}$ and *n* in Figure 12.

As to the bit-flip channel $a_{x}=1$, the results are the same as above with exchanging the roles of bases {|0〉,|1〉} and {|+〉,|−〉}. That is, $N_{\left(n=2\right)}^{T}⩾N_{\left(n=2\right)}$ for any case, $N_{\left(n⩾3\right)}^{T}<N_{\left(n⩾3\right)}$ for $N_{i}=1$, and $N_{\left(n⩾3\right)}^{T}>N_{\left(n⩾3\right)}$ for $N_{i}<N_{t}$, or $N_{t}<N_{i}<1$ but $p>p_{t}$.

**Figure 13:**
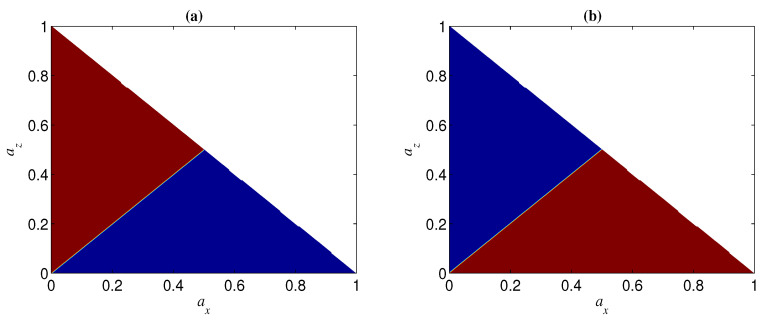
Regions of ΔN<0 (blue) and ΔN>0 (red) when $N_{i}=0.9$ and p=0.1. (**a**) n=2; (**b**) n=3.

**Figure 14:**
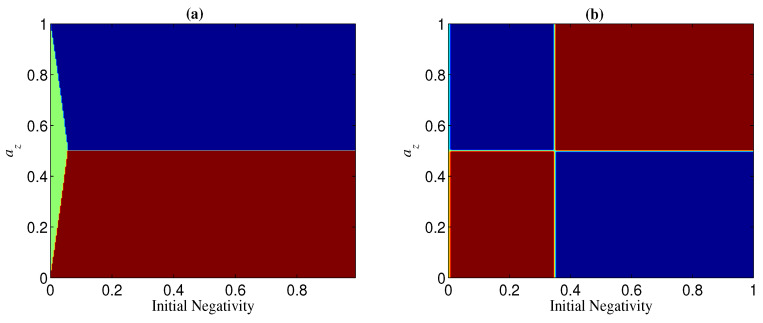
Regions of ΔN<0 (blue), ΔN>0 (red), and ΔN=0 (green) when $a_{y}=0$ and p=0.1. (**a**) n=2; (**b**) n=3.

**Figure 15:**
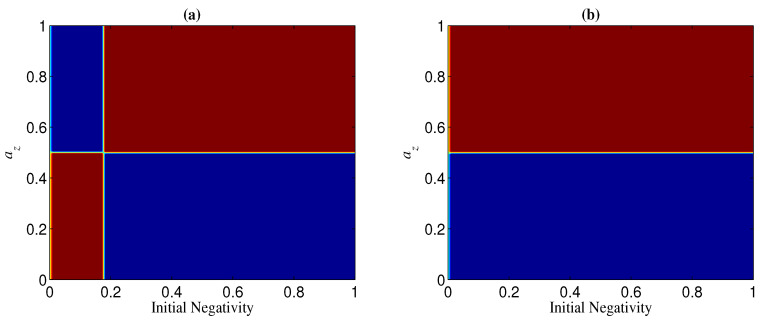
Regions of ΔN<0 (blue) and ΔN>0 (red) when $a_{y}=0$ and p=0.1. (**a**) n=4; (**b**) n=25.

In the case of $a_{z}$ and $a_{x}$ being less than one, $a_{z}=a_{x}$ is the dividing line between the two regions of ΔN>0 and ΔN<0 (see, e.g., Figure 13). Specifically, the region of ΔN>0 can be obtained by mirroring that of ΔN<0 in $a_{z}=a_{x}$ (see, e.g., Figure 13). This can be understood from the fact that the role of $a_{z}$ in $N^{T}$ is the same as that of $a_{x}$ in N, as mentioned above. From Figure 13, we can also see that $a_{z}$ ($a_{x}$) may have the opposite effect in $\Delta N_{\left(n=2\right)}$ and $\Delta N_{\left(n=3\right)}$. In other words, for given $a_{z}$ and $a_{x}$, if ΔN>0 for a two-qubit entangled state, ΔN<0 may happen for a three-qubit entangled state. This implies that the sign of ΔN is related to the number of qubits *n*. Moreover, the sign of ΔN may also be dependent on the initial entanglement $N_{i}$. We next analyze the change of the sign of ΔN with $a_{z}$ and $N_{i}$ for given *p* and $a_{y}$ (keep in mind that $a_{x}=1-a_{z}-a_{y}$, and that the noisy operator $\sigma_{y}$ has the same effect on a qubit under both bases {|+〉,|−〉} and {|0〉,|1〉}). In Figure 14, we show the sign of ΔN with different $N_{i}$ by taking $a_{y}=0$ and p=0.1. Figure 14a indicates that $\Delta N_{\left(n=2\right)}⩾0$ when $a_{z}<\left(1-a_{y}\right)/2$ and $\Delta N_{\left(n=2\right)}⩽0$ when $a_{z}>\left(1-a_{y}\right)/2$. Figure 14b, however, indicates that the sign of $\Delta N_{\left(n=3\right)}$ is dependent on the value of $N_{i}$ for a given $a_{z}$, i.e., when $N_{i}$ is larger than a threshold $N_{t}$, $\Delta N_{\left(n=3\right)}<0$ for $a_{z}<\left(1-a_{y}\right)/2$ and $\Delta N_{\left(n=3\right)}>0$ for $a_{z}>\left(1-a_{y}\right)/2$, and otherwise $\Delta N_{\left(n=3\right)}>0$ for $a_{z}<\left(1-a_{y}\right)/2$ and $\Delta N_{\left(n=3\right)}<0$ for $a_{z}>\left(1-a_{y}\right)/2$. Generally, the larger *n* is, the smaller $N_{t}$ is, and $N_{t}\to 0$ when *n* is very large (see, e.g., Figure 15).

## 3. Conclusions

In summary, we investigated the decoherence features and entanglement-robustness of cat-like states in the local Pauli noises where the probabilities of three kinds of errors occurring are not the same. It was shown that the decay law of a cat-like state is not only related to the overall noise strength (*p*), but also to the error distribution parameters $\left(a_{x},a_{y},a_{z}\right)$, and that the robustness of both two-qubit and multi-qubit states in a given noisy environment is dependent on the basis of qubits. Whether a two-qubit entangled state is more robust in the computational basis {|0〉,|1〉} or transversal basis {|+〉,|−〉} only depends on the error distribution parameters. Specifically, in terms of the entanglement-robustness of two-qubit states, the basis {|+〉,|−〉} is superior to {|0〉,|1〉} when $a_{z}<\left(1-a_{y}\right)/2$ and {|0〉,|1〉} is superior to {|+〉,|−〉} when $a_{z}>\left(1-a_{y}\right)/2$. However, which basis can be used to enhance the entanglement-robustness of multi-qubit states is not only dependent on the error distribution parameters, but also on the overall noise strength, the initial degree of entanglement, and the number of qubits. In other words, the better basis for a multi-qubit state may be not the same in different noisy environments, and the better basis for two multi-qubit states may be different in the same noisy environment. These phenomena also lead to another interesting result that the better basis for a two-qubit state and a multi-qubit state with the same degree of bipartite entanglement may be different. That is to say, if a two-qubit state is more robust under the basis {|0〉,|1〉}, a multi-qubit state with the same amount of bipartite entanglement may be more robust under the basis {|+〉,|−〉}. In addition, an *M*-qubit cat-like state could be more robust than a *L*-qubit (M>L) cat-like state having the same superposition coefficients with the former under the same qubit basis, although each qubit suffers from a Pauli noise.

The aforementioned results tell us that one could improve the robustness of cat-like entangled states in local Pauli noises by simply transforming the qubit basis. In some scenarios, one can change the basis, according to preestimated channel features, before the qubits undergoing decoherence. In other scenarios, it may be necessary to change the basis during the process of decoherece. Practically, when we should transform the basis depends on the fact of whether the better basis is related to the noise strength *p* characterizing the decoherence time. This enhancement method is much simpler and more efficient than the others because it does not introduce extra particles and works in a deterministic manner. Due to the inherent relationship between quantum entanglement and quantum coherence [57], it may be interesting to investigate whether or how the basis rotation could contribute to the preservation of quantum coherence that has attracted attention recently [58,59].

## Appendix A

For convenience, we define notations $j=j_{1}j_{2}\cdots j_{n}$ and $\bar{j}={\bar{j}}_{1}{\bar{j}}_{2}\cdots {\bar{j}}_{n}$, where $j_{m}\in \left\{0,1\right\}$ and ${\bar{j}}_{m}=1-j_{m}$ (m=1,2,⋯,n). Under the basis {|j〉,j=0,⋯,1}, the decohered state $M\left(|\Phi_{n}^{+}\rangle \right)$ can be denoted by a X matrix ϱ with diagonal and anti-diagonal elements

(A1) $$\begin{array}{c} \varrho_{j,j}=A^{n-|j|}B^{\left|j\right|}\alpha^{2}+A^{\left|j\right|}B^{n-|j|}\beta^{2}, \end{array}$$

(A2) $$\begin{array}{c} \varrho_{j,\bar{j}}=\left(C^{n-|j|}D^{\left|j\right|}+C^{\left|j\right|}D^{n-|j|}\right)\alpha \beta , \end{array}$$

where |j| denotes the number of “one” in the bit string j, e.g., |0|=0 and |1|=n. A,B,C,D are defined in Equations (11)–(14), respectively. Without loss of generality, we consider the bipartition “qubit one versus the rest”, and introduce a notation $j=j_{1}j^{\prime }$ with $j^{\prime }=j_{2}j_{3}\cdots j_{n}$. Then, the elements of the partial transpose $\varrho^{T}$ of ϱ are $\varrho_{j_{1}j^{\prime },j_{1}j^{\prime }}^{T}=\varrho_{j_{1}j^{\prime },j_{1}j^{\prime }}$ and $\varrho_{j_{1}j^{\prime },{\bar{j}}_{1}\bar{j^{\prime }}}^{T}=\varrho_{{\bar{j}}_{1}j^{\prime },j_{1}\bar{j^{\prime }}}$. Calculation of the eigenvalues of the $2^{n}\times 2^{n}$-dimensional X matrix $\varrho^{T}$ is essentially equivalent to diagonalizing $2^{n-1}$ matrices of dimension 2×2, given by

(A3) $$\left(\begin{array}{cc} \varrho_{0j^{\prime },0j^{\prime }} & \varrho_{1j^{\prime },0\bar{j^{\prime }}}\\ \varrho_{0\bar{j^{\prime }},1j^{\prime }} & \varrho_{1\bar{j^{\prime }},1\bar{j^{\prime }}} \end{array}\right)$$

with $|j^{\prime }|$ ranging from 0 to n−1. The eigenvalues of these 2×2-dimensional matrices are given by

(A4) $$\begin{array}{c} \lambda_{\pm }^{|j^{\prime }|}=\frac{1}{2}\left(\varrho_{0j^{\prime },0j^{\prime }}+\varrho_{1\bar{j^{\prime }},1\bar{j^{\prime }}}\right)\pm \frac{1}{2}\sqrt{{\left(\varrho_{0j^{\prime },0j^{\prime }}-\varrho_{1\bar{j^{\prime }},1\bar{j^{\prime }}}\right)}^{2}+4\varrho_{1j^{\prime },0\bar{j^{\prime }}}\varrho_{0\bar{j^{\prime }},1j^{\prime }}}. \end{array}$$

It can be directly calculated that

(A5) $$\;\begin{array}{ccc} \varrho_{0j^{\prime },0j^{\prime }}+\varrho_{1\bar{j^{\prime }},1\bar{j^{\prime }}} & = & A^{|j^{\prime }|}B^{n-|j^{\prime }|}+A^{n-|j^{\prime }|}B^{|j^{\prime }|}, \end{array}$$

(A6) $$\;\begin{array}{ccc} \varrho_{0j^{\prime },0j^{\prime }}-\varrho_{1\bar{j^{\prime }},1\bar{j^{\prime }}} & = & \left(A^{n-|j^{\prime }|}B^{|j^{\prime }|}-A^{|j^{\prime }|}B^{n-|j^{\prime }|}\right)\sqrt{1-N_{i}^{2}}, \end{array}$$

(A7) $$\begin{array}{ccc} 4\varrho_{1j^{\prime },0\bar{j^{\prime }}}\varrho_{0\bar{j^{\prime }},1j^{\prime }} & = & {\left(C^{n-1-|j^{\prime }|}D^{1+|j^{\prime }|}+C^{1+|j^{\prime }|}D^{n-1-|j^{\prime }|}\right)}^{2}N_{i}^{2}, \end{array}$$

where identity $|j^{\prime }\left|+\right|\bar{j^{\prime }}|=n-1$ has been used. Using the definition of negativity given in Equation (3), we have

(A8) $$\begin{array}{ccc} N & = & \sum_{|j^{\prime }|=0}^{n-1}\left(\begin{array}{c} n-1\\ |j^{\prime }| \end{array}\right)\max\left(0,-2\lambda_{-}^{|j^{\prime }|}\right)\\ & = & \sum_{|j^{\prime }|=0}^{\left\lfloor \frac{n-1}{2}\right\rfloor }\left(\begin{array}{c} n-1\\ |j^{\prime }| \end{array}\right)\max\left(0,-2\lambda_{-}^{|j^{\prime }|}\right)+\sum_{|j^{\prime }|=\left\lfloor \frac{n-1}{2}\right\rfloor +1}^{n-1}\left(\begin{array}{c} n-1\\ |j^{\prime }| \end{array}\right)\max\left(0,-2\lambda_{-}^{|j^{\prime }|}\right)\\ & = & \sum_{u=0}^{\left\lfloor \frac{n-1}{2}\right\rfloor }\left(\begin{array}{c} n-1\\ u \end{array}\right)\max\left(0,H_{u}-F_{u}\right)+\sum_{u=0}^{\left\lfloor \frac{n-1}{2}\right\rfloor }\left(\begin{array}{c} n-1\\ u \end{array}\right)\max\left(0,G_{u}-F_{u+1}\right), \end{array}$$

where $\left\lfloor \frac{n-1}{2}\right\rfloor =\left(n-1\right)/2$, for *n* being odd, or $\lfloor \frac{n-1}{2}\rfloor =n/2-1$, for *n* being even, and $F_{u},G_{u},H_{u}$ are defined in Equations (8)–(10), respectively.
